# Efficacy and Safety of Pharmacoinvasive Strategy Compared to Primary Percutaneous Coronary Intervention in the Management of ST-Segment Elevation Myocardial Infarction: A Prospective Country-Wide Registry

**DOI:** 10.5334/aogh.2632

**Published:** 2020-02-05

**Authors:** Mohammad Zubaid, Haitham Khraishah, Barrak Alahmad, Wafa Rashed, Mustafa Ridha, Fahad Alenezi, Mohamad Aljarralah, Khalid Al-Marri, Mohammad Almutairi, Khalid Althalji, Abdulhamied Alfaddagh

**Affiliations:** 1 Department of Medicine, Faculty of Medicine, Kuwait University, KW; 2 Department of Medicine, Beth Israel Deaconess Medical Center, Harvard Medical School, Boston, MA, US; 3 Environmental Health Department, T.H. Chan School of Public Health, Harvard University, Boston, MA, US; 4 Division of Cardiology, Department of Medicine, Mubarak Alkabeer Hospital, Ministry of Health, KW; 5 Department of Cardiology, Salman Aldabous Cardiac Centre, Ministry of Health, KW; 6 Division of Cardiology, Department of Medicine, Alfarwaniya Hospital, Ministry of Health, KW; 7 Department of Cardiology, Sabah Alahmad Cardiac Centre, Ministry of Health, KW; 8 Department of Cardiology, Chest Diseases Hospital, Ministry of Health, KW; 9 Division of Cardiology, Department of Medicine, Aljahra Hospital, Ministry of Health, KW; 10 Ciccarone Center for Prevention of Cardiovascular Disease, Johns Hopkins University, Baltimore, Maryland, US

## Abstract

**Background:**

A pharmacoinvasive reperfusion strategy is recommended for ST-elevation myocardial infarction (STEMI) patients when primary percutaneous coronary intervention (PCI) cannot be achieved in a timely fashion. This is based on a limited number of trials. The effectiveness of this strategy in the real-world is unclear.

**Objectives:**

To compare the effectiveness of pharmacoinvasive strategy versus primary PCI using a nationwide prospective registry of STEMI patients.

**Methods:**

We examined 936 STEMI patients from the reperfusion in ST-elevation myocardial infarction in Kuwait (REPERFUSE Kuwait) registry who underwent either primary PCI or pharmacoinvasive reperfusion. A composite outcome was measured based on death, congestive heart failure, reinfarction or stroke prospectively ascertained during hospital stay and up to one-year follow-up. The association between reperfusion strategy and the composite outcome was assessed using multivariate regression and Poisson proportional hazard model.

**Results:**

Compared to the pharmacoinvasive group, those undergoing primary PCI had higher Killip class on presentation and required more blood transfusions during hospitalization. There was no significant difference between primary PCI and pharmacoinvasive strategy with regards to the incidence of the composite outcome during the in-hospital period (RR = 1.0; 95% CI 0.98–1.02; p = 0.96) after adjustment for possible confounders. Over one-year follow-up, the survival of the two groups was not different (p = 0.66). The incidence of major bleeding was similar in both groups.

**Conclusion:**

STEMI patients treated with a pharmacoinvasive strategy have comparable outcomes to those treated with primary PCI with no increased risk of major bleeding. These real-world data support the use of a pharmacoinvasive strategy when primary PCI cannot be achieved in a timely fashion.

## Introduction

ST-segment elevation myocardial infarction (STEMI) accounts for 25–40% of acute coronary syndrome (ACS) cases [1,2,3,4]. Several studies and practice guidelines have demonstrated the superiority of primary percutaneous coronary intervention (PCI) over other therapies when performed within 90 minutes of first medical contact (FMC) for field transfer and 120 minutes of FMC for patients presenting to non-PCI-capable facility [3,5]. However, some of this superiority is lost when door-to-balloon (D2B) time exceeds 120 minutes, a situation that can occur when challenging conditions like shortage of skilled manpower, weather, traffic and geography exist [6,7,8].

Pharmacoinvasive (PhI) strategy, a reperfusion strategy that entails administration of fibrinolytic agent followed by early angiography and PCI, has been advocated as an alternative strategy to delayed primary PCI in settings where primary PCI cannot be undertaken in a guideline-recommended time frame [8,9]. The aim of this study was to evaluate the applicability, safety and 12-month clinical outcomes of PhI strategy when compared to primary PCI across an entire country where several challenges to the performance of primary PCI existed.

## Methods

### Study Design

The use of reperfusion in ST-elevation myocardial infarction in Kuwait (REPERFUSE Kuwait) is a prospective, multicenter, cohort-based registry of all patients who presented with acute ST-segment elevation myocardial infarction (STEMI), specifically examining the reperfusion strategies they received. STEMI was defined as ST segment elevation ≥1 mm in two contiguous leads on a 12-lead electrocardiogram. STEMI-patients were recruited between September 2014 and September 2015 in all hospitals in the state of Kuwait with a follow-up period of one year. The study received ethical approval from the Kuwait Ministry of Health’s ethics committee for the protection of human subjects. REPERFUSE Kuwait registry adhered to the recommendations from the “Strengthening the Reporting of observational studies in Epidemiology (STROBE) statement” on improving the quality of reporting of observational studies [10].

### Participating Hospitals And Study Population

Kuwait is a Middle Eastern country with a reported population of 3.9 million in 2015 [11]. Kuwait has six large general hospitals that belong to the public sector under the management of the Ministry of Health, in addition to a specialized tertiary cardiac center. For the duration of the study, the three STEMI-reperfusion strategies available were either (a) primary PCI in hospitals with an onsite catheterization laboratory facility (b) immediate fibrinolytic therapy alone or (c) PhI with immediate fibrinolytic therapy and subsequent coronary angiography within 24 hours of fibrinolysis. The choice of reperfusion strategy depended on the hospital of presentation and the practice in that hospital. Hospitals with onsite catheterization laboratory elected to provide primary PCI, while hospitals without onsite catheterization laboratory elected to use either PhI strategy or fibrinolysis only (Supplemental Table 1). In brief, patients presenting to Amiri and Adan hospitals received primary PCI, while those who presented to Mubarak Al-Kabeer and received PhI. Patients presented to Farwaniya and Sabah hospitals received either primary PCI or PhI, depending on the time of presentation (Supplemental Table 1). STEMI patients presenting to Al-Jahra hospital received thrombolytic therapy only and were excluded from the analysis. Patients in the PhI arm received either tenecteplase or reteplase. During hospitalization, patients received aspirin, P2Y12 inhibitor, ACEi/ARB, beta blockers and a statin therapy (Supplemental Table 2). Patients aged 65 years and older received half dose of fibrinolytic therapy (whether tenecteplase or reteplase), if they were planned for PhI strategy.

### Data Collection

We prospectively collected data using a standardized case report form (CRF). Data variables were in accordance with American College of Cardiology (ACC) key data elements and definitions for measuring the clinical management and outcomes of patients with acute coronary syndrome (ACS) [12]. Incident cases were enrolled on a daily basis for the duration of the study. Critical times in CRFs were measured using ambulance reports, emergency department forms, ECG papers, and catheterization laboratory reports. Follow-up was planned at 1, 6 and 12 months from the date of enrolment. Follow-up was carried out by clinic visit or telephone interview.

### Study Outcomes

The outcome of the study was a composite of death, congestive heart failure, reinfarction or stroke within hospitalization and at one year. Congestive heart failure was defined by the development of symptoms, signs or radiological evidence of pulmonary edema/congestion requiring diuretic therapy. Reinfarction was defined as recurrent signs and symptoms of ischemia at rest, accompanied by new or recurrent ST-segment elevations of ≥0.1 mV in at least two contiguous leads lasting ≥30 minutes. Stroke was defined as rapidly developing clinical signs of focal (or global) disturbance of cerebral function, with symptoms lasting 24 hours or longer or leading to death, with no apparent cause other than of vascular origin [13]. The Bleeding Academic Research Consortium (BARC) criteria was used to classify bleeding types [14]. Major bleeding was defined as BARC type 2 or higher, that is any overt sign of hemorrhage that is actionable and requires diagnostic studies, hospitalization or treatment by health care professional [14].

### Statistical Analysis

We summarized continuous variables by their means (standard deviation), time-related continuous variables by their medians (interquartile range), and categorical variables by absolute numbers (percentages). We tested for difference in means between the two intervention groups using 2-samples t-test, and difference in medians using Wilcoxon rank-sum (2 groups) or Kruskal-Wallis (>2 groups) tests. Fisher’s exact test was used for the categorical variables.

We fitted log-Poisson models with binary outcome (in-hospital composite outcome: yes/no) to obtain relative risks comparing the two intervention groups. We used robust standard errors to correct the inferences of the Poisson model coefficients. Multivariate models were used to adjust for a number of covariates. Choice of covariate inclusion to control for confounding was based on *a priori* clinical hypotheses. Therefore, all models were adjusted for individual characteristics: age (linear) and gender, severity indicators: time from symptom onset to hospital (>3 hours vs. <3 hours), Killip score (I vs. II-IV), MI location (anterior vs. other), previous history: smoking (recent/current vs. not), diabetes (yes/no) or dyslipidemia (yes/no), and clinical indicators on admission: BMI (linear), heart rate (linear), and systolic blood pressure (linear). We checked the fitness of our models using Poisson goodness-of-fit tests. To test if the in-hospital composite outcome from a given exposure differs across the two intervention groups, we did a stratified subgroup analysis to check for effect measure modification on the multiplicative scale between intervention strategy and other covariates of interest.

From the time of hospital discharge for each individual and up to one year, we created a time-to-event composite outcome and a censoring variable to account for loss of follow-up. Data on event times were only available arbitrarily at the time of interview/phone call (1, 6, and 12 months). We sorted the data into time intervals and calculated the person-time contributed by each individual. We assumed that events and censorings are uniformly distributed throughout the time interval (contributing half the person-time). We fitted a Poisson regression to model the rate of events in the time category using person-days contributed in that category as an offset. To test the difference between the two intervention groups in the incidence rate ratio across all times, we used the p-value from the Wald test that corresponds to the group coefficient. We also tested whether the survival of intervention groups varied over time.

All p-values were two-sided with a significance level of less than 0.05. Data analyses were conducted using Stata/IC v.15.0 (StataCorp LLC).

## Results

**Figure 1:**
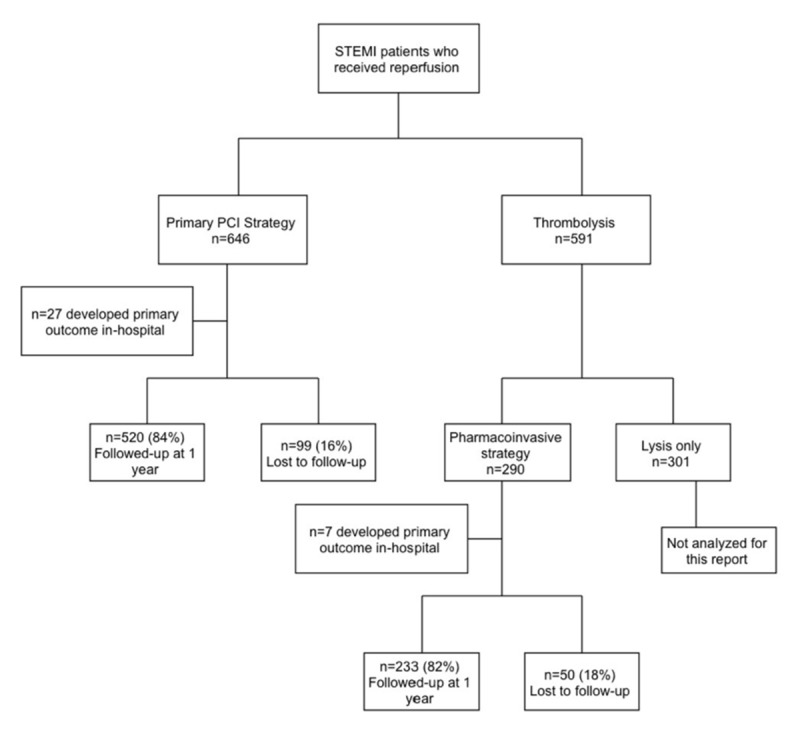
Flowchart of Patient Recruitment. A total of 1,237 STEMI patient were included in all-inclusive registry between September 2014 and September 2015; 646 patients underwent primary PCI with follow-up of 84% at one year. 290 patients underwent PhI with one year follow-up of 82%. We excluded 301 subjects who received lysis therapy only.

Between September 2014 and September 2015, we enrolled 1,237 patients who received reperfusion therapy for a diagnosis of STEMI. Of those, 646 patients (52.2%) underwent primary PCI and 290 patients (23.4%) had PhI therapy with 22 patients undergoing rescue PCI after failed lytic therapy. We excluded 301 patients (24.3%) from this analysis as they underwent fibrinolysis therapy only. Over the study period, loss to follow-up was balanced between primary PCI and PhI groups (16% vs. 17.7%, respectively) (Figure 1). There was higher loss to follow-up over the entire study period among non-nationals compared to Kuwaiti nationals (p = 0.002).

Baseline characteristics are shown in Table 1. The mean age was 52.3 ± 10.2 years for the PhI group and 53.7 ± 10.2 years for the primary PCI group. The majority of patients in the study sample were males (93.7% of the primary PCI group and 94.1% of the PhI group). We found no significant difference between the two groups with regards to age, gender distribution, prevalence of prior PCI, prior coronary artery bypass surgery, prior myocardial infarction, or common cardiovascular risk factors including obesity, hypertension, diabetes mellitus, or dyslipidemia. STEMI location was similar between the two groups with anterior infarction being the most common. Compared to the PhI group, patients in the primary PCI group were more likely to have a higher Killip class on admission and a personal history of stroke.

**Table 1:** Patients Baseline Characteristics.

Characteristic	Primary PCI (n = 646)	PhI (n = 290)	p-value

Age			
Overall (years), mean (SD)	53.7 (10.2)	52.3 (10.2)	0.055
≥ 75 year, n (%)	20 (3.1%)	10 (3.4%)	
Female sex, n (%)	41 (6.3%)	17 (5.9%)	0.88
Weight (kg), mean (SD)	78.9 (14.5)	80.4 (14.0)	0.16
BMI (kg/m^2^), mean (SD)	27.7 (4.6)	27.7 (4.4)	0.96
Heart Rate (beats/min), mean (SD)	83.1 (19.4)	79.3 (17.7)	0.005
Systolic BP (mmHg), mean (SD)	136.7 (29.5)	135.3 (28.6)	0.56
Creatinine (mg/dl), mean (SD)	1.0 (0.6)	0.9 (0.2)	0.01
Hemoglobin (g/dl), mean (SD)	15.3 (7.3)	15.4 (8.5)	0.89
Killip Class I at Time of Arrival, n (%)	575 (89.0%)	275 (95.2%)	0.001
Anterior MI, n (%)	342 (52.9%)	143 (50.7%)	0.53
Time from symptom onset to hospital arrival <3 hours, n (%)	452 (70.0%)	209 (72.1%)	0.54
Risk factors, n (%)			
Previous PCI	62 (9.6%)	21 (7.2%)	0.26
Previous CABG	7 (1.1%)	3 (1.0%)	0.99
Previous MI	73 (11.3%)	32 (11.1%)	0.99
Peripheral Arterial Disease	9 (1.4%)	2 (0.7%)	0.52
Previous Stroke	20 (3.1%)	1 (0.3%)	0.007
Hypertension	249 (38.5%)	112 (38.6%)	0.99
Diabetes Mellitus	225 (34.8%)	92 (31.7%)	0.37
Dyslipidemia	181 (28.0%)	73 (25.2%)	0.38
Current or Recent Smoker	325 (50.3%)	140 (48.3%)	0.57

CABG = coronary artery bypass graft; MI = myocardial infarction; PCI = percutaneous coronary intervention; PhI = pharmacoinvasive; SD = standard deviation.

Key time intervals are presented in Table 2. The median times from symptom onset to first hospital arrival and to first ECG were similar among the study groups. The median door-to-balloon time among patient presenting to primary PCI facility was 58 minutes (IQR: 40 to 84 min) and 99.5 min (IQR: 79 to 137 min) in patients with interhospital transfer for primary PCI. Patients in the PhI group had a median door-to-needle time of 34 min (IQR: 23 to 55 min) and fibrinolysis to catheterization time of 16.6 hours (IQR: 12.5 to 27 hours). However, 56 patients (19.3%) in the PhI group underwent catheterization after more than 24 hours from receiving thrombolytic therapy.

**Table 2:** Key Time Intervals in primary PCI and pharmacoinvasive groups.

	Unit	Primary PCI (n = 646) Median [IQR]	PhI (n = 290) Median [IQR]	p-value

Time from symptom onset to first hospital arrival	*Minute*	115 [60, 201]	113 [60, 196]	0.67
Time from hospital arrival to first ECG	*Minute*	5 [4, 10]	12 [7, 19]	<0.001
Door-to-balloon time among all primary PCI group	*Minute*	68 [45, 100]	NA	NA
*Patients presenting to primary PCI facility n = 482)*	*Minute*	58 [40, 84]	NA	NA
*Patients presenting to non-primary PCI facility (n = 150)*	*Minute*	99.5 [79, 137]	NA	NA
Door-to-needle time	*Minute*	NA	35 [23, 55]	NA
Time from administration of fibrinolytic therapy to catheterization among all PhI group	*Hours*	NA	16.6 [9.5, 22.5]	NA
Time from symptom onset to catheterization lab	*Hour*	3.3 [2.2, 5.1]	19 [12.5, 27.0]	NA

IQR = interquartile range; NA = not applicable; PCI = percutaneous coronary intervention; PhI = pharmacoinvasive.

Table 3 shows the details of the procedures performed. PCI was performed in 98.4% in primary PCI group compared to 80.6% in PhI group (p < 0.001) with left anterior descending (LAD) artery being the most common culprit artery among both groups. Radial access was used in 54.9% of patients who underwent primary PCI compared to 75% of patients undergoing PCI in the PhI group.

**Table 3:** Details of Procedures Performed.

	Primary PCI (n = 646)	PhI (n = 290)	p-value

PCI Performed	635/646 (98.4%)	233/288 (80.6%)	<0.001
Stent(s) Placed	613/643 (95.3%)	229/273 (83.9%)	<0.001
Access Site			
Brachial	1 (0.2%)	0 (0.0%)	<0.001
Femoral	290 (45.0%)	72 (25.0%)	
Radial	354 (54.9%)	216 (75.0%)	
Access Site Complications			
Hematoma	12 (1.9%)	0 (0%)	0.023
Occlusion	2 (0.3%)	0 (0%)	0.99
Pseudoaneurysm	1 (0.2%)	1 (0.3%)	0.52
Peripheral embolization	0	0	NA
AV fistula	0	0	NA
Culprit artery			
LM	6 (0.9%)	2 (0.7%)	0.06
LAD	342 (53.1%)	142 (49.1%)	
Circumflex	86 (13.4%)	34 (11.8%)	
RCA	197 (30.6%)	95 (32.9%)	
Others	13 (2.0%)	16 (5.5%)	

AV = arteriovenous; LAD = left anterior descending; LM = left main; NA = not applicable; PCI = percutaneous coronary intervention; PhI = Pharmacoinvasive; RCA = right coronary artery.

During hospital stay, the primary composite end point of death, congestive heart failure, reinfarction or stroke occurred in 27 patients (4.1%) in primary PCI group and seven patients (2.4%) in PhI group (p-value = 0.12) (Table 4). The incidence of individual outcomes was similar between both groups. The incidence of bleeding was similar in both groups. Notably, patients in the primary PCI group had more blood transfusions compared to the PhI group (1.7% vs. 0%, respectively, p-value = 0.02).

**Table 4:** In-hospital and at Follow-up Outcomes.

	Primary PCI n/N (%)	PhI n/N (%)	p-value

In-hospital composite Outcome of death, reinfarction, stroke, or CHF	27/646 (4.2%)	7/290 (2.4%)	0.12
Death	11/646 (1.7%)	3/290 (1.0%)	0.57
Reinfarction	7/645 (1.1%)	3/285 (1.1%)	1.00
Stroke	2/634 (0.3%)	0/279 (0%)	1.00
CHF	10/597 (1.7)	1/271 (0.4%)	0.19
Any Bleeding	13/646 (2.0%)	4/290 (1.4%)	0.61
Major Bleeding*	10/646 (1.5%)	2/290 (0.7%)	0.36
Transfusion	11/646 (1.7%)	0/290 (0%)	0.02
Composite outcome: death, reinfarction, stroke or CHF at follow up			
1 month	16/595 (2.9%)	3/260 (1.2%)	0.77
6 months	11/537 (2.0%)	6/244 (2.5%)	0.79
12 months	16/493 (3.3%)	8/224 (3.6%)	0.83
Total loss to follow-up (over study period)	99/619 (16%)	50/283 (17.7%)	0.56

CHF = congestive heart failure; PCI = percutaneous coronary intervention; PhI = Pharmacoinvasive. * Any overt sign of hemorrhage that is actionable and requires diagnostic studies, hospitalization or treatment by health care professional.

**Figure 2:**
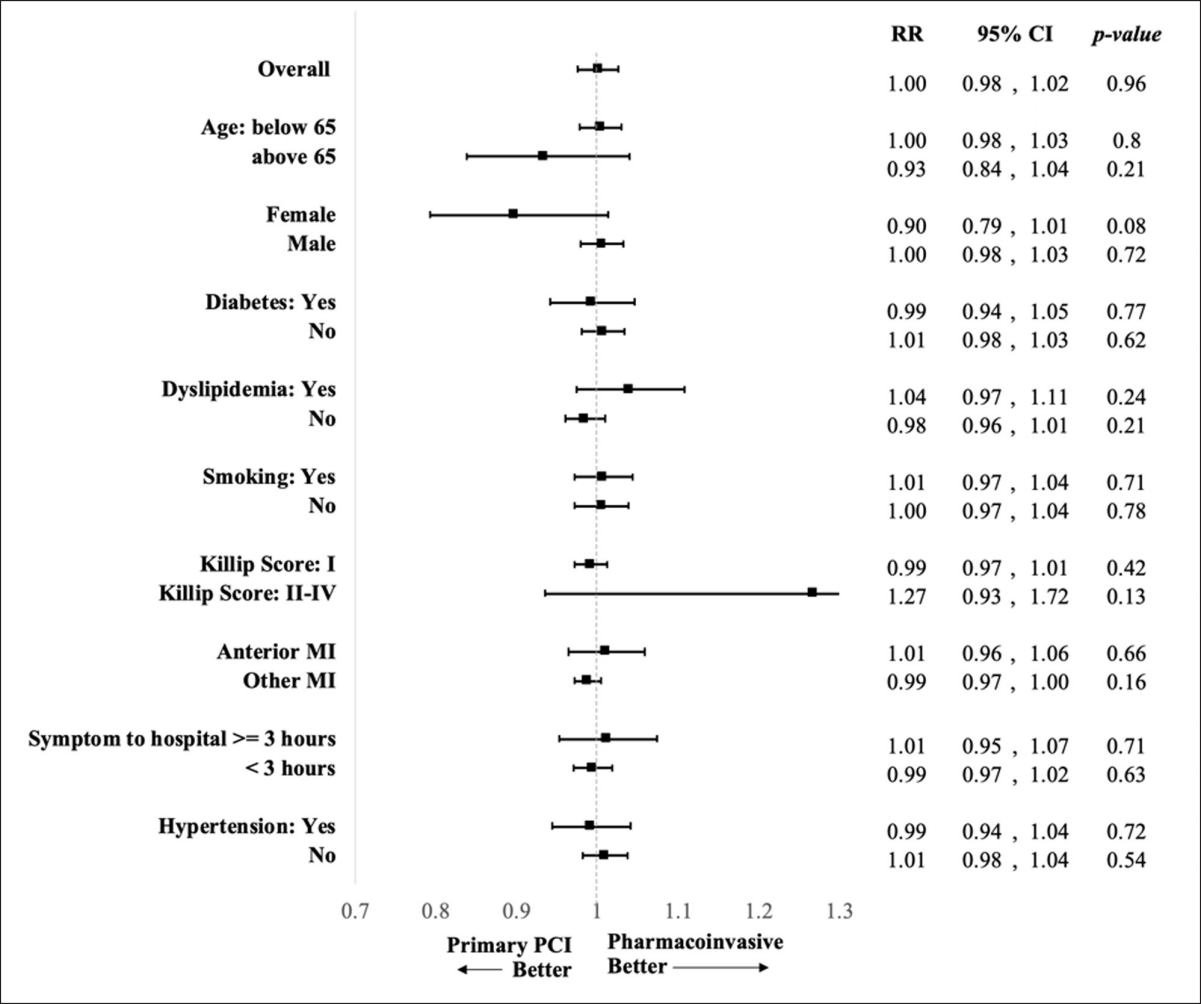
Subgroup analyses for the risk of developing in-hospital composite outcome comparing the two interventions. There was no difference between primary PCI and PhI in terms of in-hospital composite outcome (death, reinfarction, CHF and stroke) in the overall group and subgroup analysis using following categories: age, sex, diabetes, dyslipidemia, smoking status, Killip score, MI location, duration from symptom onset to hospital arrival and hypertension. All models were adjusted for baseline systolic blood pressure, baseline heart rate and baseline BMI.

Figure 2 shows subgroup analyses of the composite end point among the two treatment groups. There was no significant difference in the relative risk of the composite outcome between both groups after adjusting for baseline systolic blood pressure, baseline heart rate, baseline BMI and time from symptom onset to hospital arrival. Compared to non-diabetics, diabetic patients were more likely to experience the composite outcome during their hospital stay (relative risk, RR = 2.13; 95% CI, 1.09–4.15, p-value = 0.026) (Supplementary Table 2). Also, a higher Killip class on presentation was associated with increased risk of developing the composite outcome, when compared to patients presenting with Killip class I (Supplementary Table 2).

**Figure 3:**
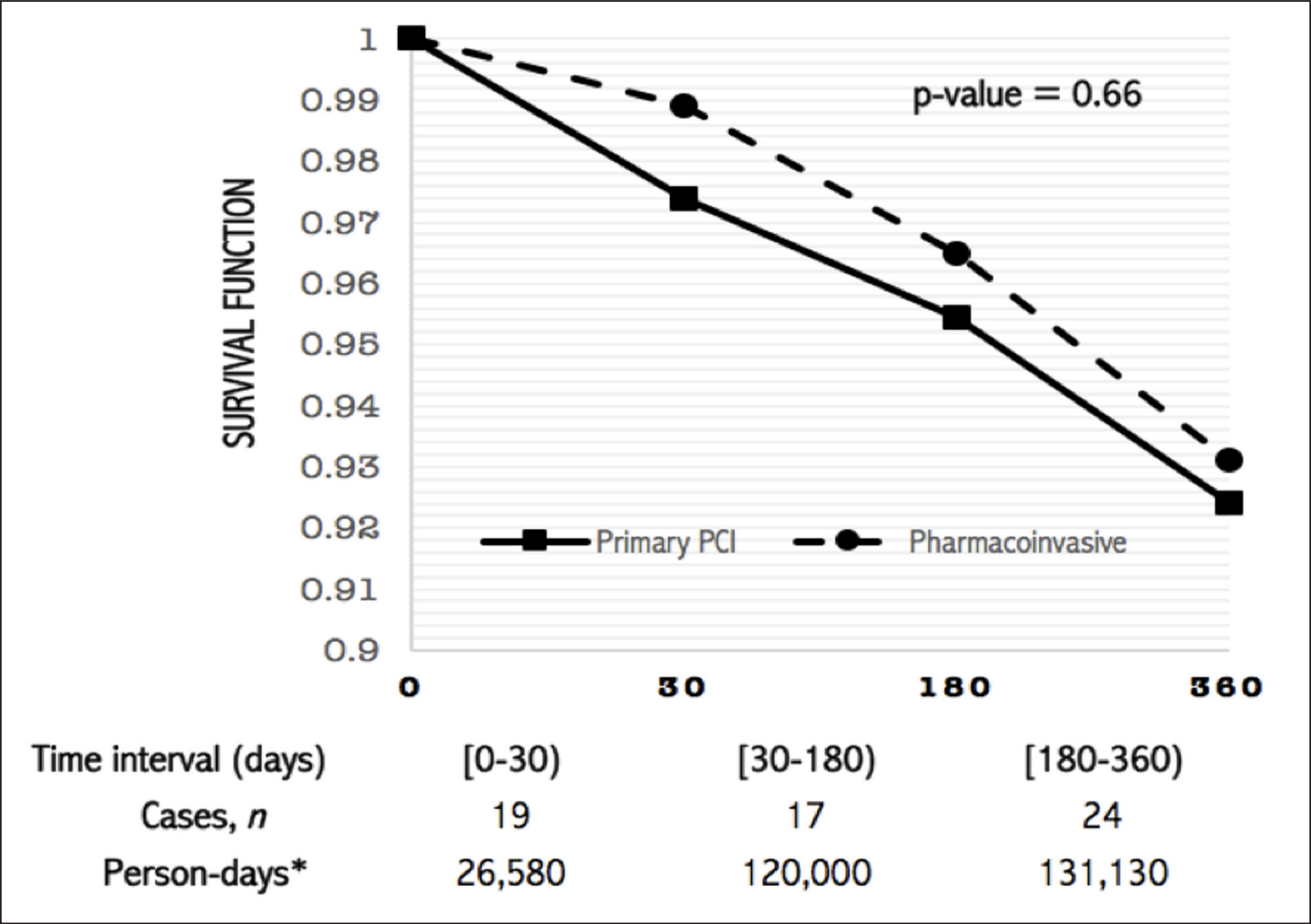
One-year follow-up: Poisson Proportional Hazard Model. No difference was found between primary PCI and PhI in terms of the incidence rate of the composite outcome at one year using survival model looking, after sorting the total of person-days contributed by each individual for three time intervals at 1, 6, and 12 months.

Follow-up for the composite outcome was done arbitrarily at 1, 6 and 12-month intervals after hospital discharge (Table 4 and Figure 3). There was no difference in the incidence of the composite outcome between both treatment arms at any given time interval (Table 4); however, the incidence rate of the composite outcome was significantly higher at one month from discharge when compared to both 6 and 12-month follow-up (Supplementary Table 3).

## Discussion

To our knowledge, this is the first prospective study in Kuwait and the Middle East to compare the outcomes of primary PCI and PhI strategies in acute STEMI in a real-world setting. We found no significant difference between primary PCI and PhI in terms of the composite endpoint of death, congestive heart failure, re-infarction and stroke during in-patient hospitalization period or at one-year follow-up. We also found no significant difference between the two treatment groups in terms of bleeding events; however, blood transfusion rates were higher in the primary PCI group when compared to PhI group.

For STEMI patients presenting within 12 hours of symptom onset, primary PCI remains the preferred method of reperfusion when door-to-balloon time can be achieved in less than 90 minutes when presenting to PCI-capable facility or less than 120 minutes when presenting to non-PCI capable facility. However, due to logistic and geographic barriers, many patients present to non-PCI capable facilities and are unable to undergo primary PCI within the time frames recommended by the guidelines [3,5]. In fact, only one third of the hospitals in the United States have the capability to perform primary PCI round the clock [15]. In that setting, PhI strategy, which entails delivering fibrinolytic therapy followed by transfer to PCI-capable facility for early PCI within 3–24 hours, seems a reasonable option. The rationale behind this approach is that the initial fibrinolytic therapy would restore coronary circulation early on. Then, invasive intervention with PCI would either reopen the culprit artery in case of failed thrombolysis or augment the outcomes of a successful fibrinolysis [16]. This strategy was tested in the STREAM trial (Strategic Reperfusion Early after Myocardial Infarction) [8]. Investigators of the STREAM trial randomized 1892 STEMI patients, who presented within three hours of symptom onset and were unable to undergo primary PCI within one hour. Patients were randomized to receive either primary PCI or PhI therapy with coronary angiography within 6–24 hours. The primary end point (a composite of death, shock, congestive heart failure or re-infarction within 30 days) was similar between the two intervention arms (12.4% in the PhI group and 14.3% in the primary PCI). At one-year follow-up, all-cause mortality and cardiac mortality were similar between treatment groups [17]. The STREAM trial provides informative insights about the comparability of PhI to primary PCI when primary PCI is not readily available. However, it was carried out in a highly controlled environment that is difficult to simulate in real-world settings, especially in developing countries, as it excluded patients presenting after three hours of symptom onset, and all patients received coronary angiography within 24 hours of fibrinolysis. Additionally, the optimal time of PCI in the PhI has not been established yet. Current evidence supports performing coronary angiogram within 3–24 hours of administering fibrinolytic therapy [3,5] as facilitated PCI within three hours of fibrinolytic therapy was associated with worse outcomes per ASSENT-4 PCI (Assessment of the Safety and Efficacy of a New Treatment Strategy with Percutaneous Coronary Intervention) trial [18].

Our study findings in a Middle Eastern population confirms the results of similarly conducted studies, namely Minneapolis Heart Institute (MHI) regional STEMI program [19]. and Ottawa health care system [20] in North American population, Korea Acute Myocardial Infarction Registry (KAMIR) [21] in Asian population and FASTMI2015 (French Registry of Acute ST-elevation or Non-ST-elevation Myocardial Infarction 2015) [22] in European population. However, several differences exist in terms of time delays and complication rate especially bleeding. In our study cohort, the median time from fibrinolysis to elective PCI in PhI was 16.6 hours with 80.7% patients receiving PCI within 3–24 hours. While the median time delays from fibrinolysis to PCI were 4 hours and 20 minutes within University of Ottawa regional STEMI system [20], and 41.5 hours in the KAMIR Registry [21] in Korea. Additionally, our study patients were younger (mean age for the total group was 53.3 ± 10.2 years, almost 8–10 years younger than STEMI patients studies in the aforementioned registries) with higher prevalence of diabetes as compared to the aforementioned registries, which might in part explain lower in hospital mortality when compared to other registries. The longer time delays in our study can be attributed to limited resources including the presence of only two around the clock catheterization laboratories and limited number of trained interventional cardiologists.

In terms of safety, the risk of bleeding was similar between both treatment arms, and the bleeding risk was low (2% in primary PCI Vs. 1.4% in PhI). No intracranial hemorrhage was observed in either treatment group. We believe low risk of bleeding was driven by two main processes: (i) the use of half dose of tenecteplase in patients aged >65, and (ii) younger mean age of patients. Before the amendment of the STREAM trial protocol, the risk of intracranial bleeding was higher in the PhI arm compared to the primary PCI arm (0.96% vs. 0.21%; p = 0.04). However, after reducing tenecteplase dose by 50% in patients older than 75 years, the risk of intracranial bleeding was similar in both arms (0.54% vs. 0.26%; p = 0.45) [8,17]. Transfusion rates were higher among the primary PCI group compared to the PhI group (1.7% vs. 0%, p-value = 0.02). Although not statistically significant, the risk of major bleed was higher in the primary PCI group (1.5%) compared to PhI group (0.7%). This is likely driven by the fact that 45% of patients in the PCI group got coronary angiography via femoral access when compared to only 25% in PhI group.

### Strength And Implications

Our study is a prospective, all-inclusive registry of STEMI patients in Kuwait with one-year follow-up. It examined the day to day practice of STEMI management in a setting where physicians utilized what is available to them in terms of facilities and infrastructure with all its limitations. Over the follow-up period, there was no competing risks because death from any cause was included in the composite outcome. The results of this analysis have significant implications on the practice of treating STEMI in Kuwait, where we have real limitations in skilled manpower and the use of emergency medical services. The shortage of skilled manpower is exaggerated during holiday seasons when even PCI-capable hospitals would not have enough staff to carry out primary PCI round the clock. The underuse of emergency medical services was observed in several of our previous local registries where less than 17% of ACS patients use ambulance services to get to the hospital [23,24]. This results in most patients presenting first to non-PCI-capable hospitals. Therefore, it is reassuring to know that we have at our disposal an effective and safe strategy of reperfusion when primary PCI is not available or can’t be performed in a timely fashion.

### Limitations

This was a registry with all the inherent biases that registries might have. For example, patients with prior stoke and patients with higher Killip class received more primary PCI strategy than PhI strategy. This might explain the numerical trend towards worse outcomes in the primary PCI group. Because we included all comers, we had a large percentage of expatriates in the study. Many non-nationals leave the country for good or for extended vacations when they suffer from ACS. This has affected the number of patients who were lost to follow-up, although this was balanced between both treatment groups. Also, we were not able to obtain exact times of events during the one-year follow-up; therefore, it was not possible to fit a cox proportional hazard model. Sorting the data into time intervals may have reduced the power to detect differences. We had to make strong assumptions on the distribution of events and censorings across the sorted data.

## Conclusions

In conclusion, using real-world nation-wide data, the short-term and long-term cardiovascular and bleeding outcomes for pharmacoinvasive reperfusion approach for patients presenting with STEMI is comparable to primary PCI. These finding strengthen the evidence for using a pharmacoinvasive approach for patient presenting to non-PCI capable hospitals when primary PCI cannot be achieved in a timely fashion.

## Clinical Perspectives

### Competence in Medical Knowledge

When reperfusion with primary PCI is not possible, a pharmacoinvasive strategy is a reasonable alternative with similar outcomes in terms of mortality, reinfarction, heart failure and stroke.

### Translational outlook

Further studies are needed to quantify the optimal time for coronary angiography after fibrinolytic therapy.

## Additional Files

The additional files for this article can be found as follows:

10.5334/aogh.2632.s1 Supplemental Table 1. Kuwait general hospitals, population served and perfusion strategy.

10.5334/aogh.2632.s2 Supplemental Table 2. In-Hospital composite outcome: Log Poisson Model.

10.5334/aogh.2632.s3 Supplemental Table 3. One year follow-up incidence rate ratio of composite outcome: Poisson Survival Model.

## Abbreviations

PCI ≡ Percutaneous coronary intervention

PhI ≡ Pharmacoinvasive

STEMI ≡ ST-segment elevation myocardial infarction
