# Supplementary material for: Efficacy and Safety of Pharmacoinvasive Strategy Compared to Primary Percutaneous Coronary Intervention in the Management of ST-Segment Elevation Myocardial Infarction: A Prospective Country-Wide Registry
Source: Ann Glob Health. 2020 Feb 5;86(1):13. doi: 10.5334/aogh.2632 (PMC7006601; doi:10.5334/aogh.2632)
Supplement: Supplemental Table 1. — Kuwait general hospitals, population served and perfusion strategy. [file agh-86-1-2632-s1.pdf]

Supplementary Material:

Supplemental Table 1: Kuwait general hospitals, population served and perfusion strategy

Hospital	Population served	Cath lab availability	Reperfusion strategy
Amiri	572,638	Available onsite	Primary PCI
Adan	1,246,659	Available onsite	Primary PCI
Mubarak Al-Kabeer	946,452	Not available	PhI
Sabah	1,174,088	Not available	7am-4pm: Interhospital transfer for primary PCI<br>4pm-7am: PhI
Farwaniya		Not available	
Jahra	555,626	Not available	Fibrinolysis only
