# Supplementary material for: Efficacy and Safety of Pharmacoinvasive Strategy Compared to Primary Percutaneous Coronary Intervention in the Management of ST-Segment Elevation Myocardial Infarction: A Prospective Country-Wide Registry
Source: Ann Glob Health. 2020 Feb 5;86(1):13. doi: 10.5334/aogh.2632 (PMC7006601; doi:10.5334/aogh.2632)
Supplement: Supplemental Table 2. — In-Hospital composite outcome: Log Poisson Model. [file agh-86-1-2632-s2.pdf]

Supplemental Table 2: In-Hospital composite outcome: Log Poisson Model

	**Unadjusted**			Adjusted			**p-value for interaction**
	RR	95% CI	p-value	RR	95% CI	p-value	
**PhI vs PCI**	0.58	0.25 – 1.31	0.189	1.11	0.46 – 2.67	0.815	
**Diabetes**	2.47	1.27 – 4.80	0.007	2.13	1.09 – 4.15	0.026	0.43
**Dyslipidemia**	1.46	0.74 – 2.92	0.278	0.88	0.50 – 1.58	0.676	0.09
**Smoking**	0.80	0.41 – 1.56	0.510	0.97	0.52 – 1.82	0.924	0.93
**Killip Score**							
**Killip II**	8.02	3.38 – 19.03	<0.001	5.39	2.16 – 13.49	<0.001	0.70
**Killip III**	16.19	6.03 – 43.45	<0.001	7.99	2.87 – 22.19	<0.001	
**Killip IV**	32.14	16.18 – 63.86	<0.001	15.59	5.51 – 44.10	<0.001	
**Ant. Infarct**	4.12	1.72 – 9.87	0.001	2.14	0.90 – 5.10	0.086	0.99
**Age (years)**	1.07	1.04 – 1.09	<0.001	1.03	1.01 – 1.06	0.019	0.57
**HR (bpm)**	1.03	1.02 – 1.04	<0.001	1.01	1.00 – 1.02	0.100	0.44
