# Supplementary material for: Efficacy and Safety of Pharmacoinvasive Strategy Compared to Primary Percutaneous Coronary Intervention in the Management of ST-Segment Elevation Myocardial Infarction: A Prospective Country-Wide Registry
Source: Ann Glob Health. 2020 Feb 5;86(1):13. doi: 10.5334/aogh.2632 (PMC7006601; doi:10.5334/aogh.2632)
Supplement: Supplemental Table 3. — One year follow-up incidence rate ratio of composite outcome: Poisson Survival Model. [file agh-86-1-2632-s3.pdf]

Supplemental Table 3: One year follow-up incidence rate ratio of composite outcome: Poisson Survival Model

	Rate Ratio	95% CI	p-value	p-value for interaction
PHI vs. PCI	0.88	0.50 – 1.54	0.655	0.209
Follow-up intervals				
1 month	Referent			
6 months	0.19	0.10 – 0.38	<0.001	
12 months	0.25	0.14 – 0.47	<0.001	
